# DeepTool: A deep learning framework for tool wear onset detection and remaining useful life prediction

**DOI:** 10.1016/j.mex.2024.102965

**Published:** 2024-09-19

**Authors:** Pooja Kamat, Satish Kumar, Ketan Kotecha

**Affiliations:** a Symbiosis Institute of Technology, Symbiosis International (Deemed University), Pune, Maharashtra, India; b Symbiosis Centre for Applied Artificial Intelligence, Symbiosis International (Deemed University), Pune, Maharashtra, India

**Keywords:** Milling, Tool-wear, Remaining useful life, Autoencoder: LSTM, LSTM Encoder-Decoder, DeepTool: A Deep Learning Framework for Tool Wear Onset Detection and Remaining Useful Life Prediction

## Abstract

Milling tool availability and its useful life estimation is essential for optimisation, reliability and cost reduction in milling operations. This work presents DeepTool, a deep learning-based system that predicts the service life of the tool and detects the onset of its wear. DeepTool showcases a comprehensive feature extraction process, and a self-collected dataset of sensor data from milling tests carried out under different cutting settings to extract relevant information from the sensor signals. The main contributions of this study are:

• Self-Collected Dataset: Makes use of an extensive, self-collected dataset to record precise sensor signals during milling.
• Advanced Predictive Modeling: Employs hybrid autoencoder-LSTM and encoder-decoder LSTM models to estimate tool wear onset and predict its remaining useful life with over 95 % R2 accuracy score.
• Comprehensive Feature Extraction: Employs an efficient feature extraction technique from the gathered sensor data, emphasising both time-domain and frequency-domain aspects associated with tool wear.

## Graphical abstract

**Figure:**
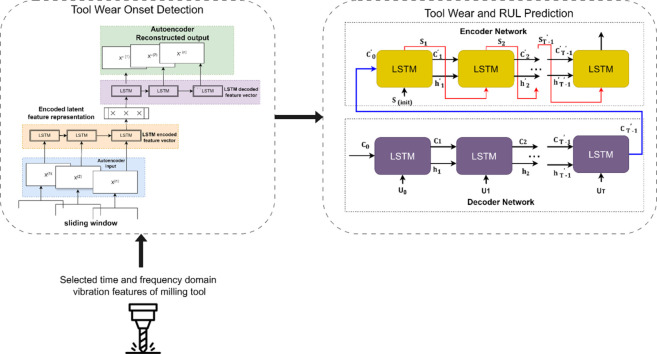

Specifications table

**Table:** 

Subject area:	Engineering
More specific subject area:	Deep Learning
Name of your method:	DeepTool: A Deep Learning Framework for Tool Wear Onset Detection and Remaining Useful Life Prediction
Name and reference of original method:	Remaining Useful-Life Prediction of the Milling Cutting Tool Using Time–Frequency-Based Features *[Sayyad, S., Kumar, S., Bongale, A., Kotecha, K., & Abraham, A. (2023). Remaining Useful-Life Prediction of the Milling Cutting Tool Using Time–Frequency-Based Features and Deep Learning Models. Sensors, 23(12), 5659*
Resource availability:	*Data: Data available on request* Software: Google Colab, Scipy library

## Background

**Fig. 1:**
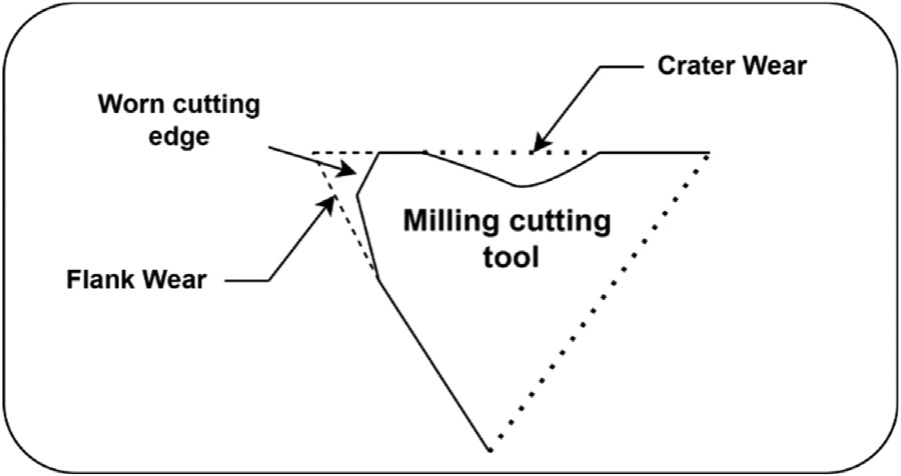
Types of tool wear faults in milling process.

Cutting tools are one of the most vital components of milling machines [1]. The main objective in milling is to make flat surfaces in different orientations, such as radial or curved surfaces. To fulfil these tasks, the workpiece is fed progressively by a spirally revolving cutter at a high or moderate speed [2]. As shown in Fig. 1, tool wear is common in the milling process. The term "tool wear" describes the slow deterioration of a tool's cutting edge during machining operations. Wear is most noticeable when cutting at low speeds. Crater wear (represented by wear and metal spreading on the tool surface) prevails at high speeds, especially when cutting ductile materials. It occurs at the tool angle due to the interaction between crater and flank wear [3,4]. Therefore, it is important for milling machine supervisors to accurately predict tool wear and RUL (Remaining Useful Life). The predictive metric RUL calculates how long a tool will last or be useful before it breaks down or becomes ineffective. Cutting tool RUL prediction systematically tracks the course of cutting tool wear, enabling proactive maintenance and replacement [5]. Th wear progress of the cutting tool follows a systematic path that can be monitored through RUL prediction for cutting tools, enabling proactive maintenance and replacement. Tool rupture can lead to significant productivity loss and catastrophic downtime. To avoid such economic loss, predictive maintenance techniques based on real-time health monitoring systems are necessary [6]. Although development in diagnostics for cutting tools has been advancing in recent years, the field of prognostics for milling machinery is still in its inception, but research is quickly taking off.

RUL prediction methods for cutting tools and milling machinery are usually divided into model-based and data-driven approaches. These physics-based or degradation models are based on an understanding of the physical processes and mechanisms involved in the degradation of systems or components. However, developing a proper degradation mathematical model for milling machinery and cutting tools in complex operating conditions is difficult. Therefore, data-driven techniques have been very popular in the field of tool wear prognostics in recent years. The methods, however, can treat complex relationships and patterns without explicit use of such understanding about the underlying degradation mechanisms [7]. However, most of the research in this domain has focused on RUL prediction during the wear-out stage of the cutting tool. Therefore, there is a dire need for research that focuses on identifying the onset of tool wear and predicting RUL from the moment tool wear is detected. Deep Learning and machine learning techniques have recently become extremely popular for solving classification and regression-based problems across various domains, including healthcare, surveillance, manufacturing, etc. [7,8].

However, most research in this domain has focused on RUL prediction within the wear-out stage of the cutting tool. The motive for the research in this study is, therefore, to develop an integrated predictive maintenance framework able to detect incipient abnormalities in milling machinery and tools, together with the prediction of tool wear and RUL by data-driven methodologies informed through deep learning.The work attempts to use a deep learning-based DeepTool framework to predict tool wear and estimate RUL using self-collected vibration data from accelerometers. This collection includes sensor data gathered while conducting testing with various milling cuts. DeepTool approach combines advanced deep learning models such as Autoencoder-LSTM (AE_LSTM) and Encoder-Decoder LSTM (E-D_LSTM) with techniques for anomaly pattern mining and wear-onset monitoring. The models are trained against the history of data containing normal and abnormal samples to capture these complex patterns associated with the degradation process. AE-LSTM models capably identify the onsets of tool wear by learning latent representations of input data through representative features from autoencoders and LSTMs [9,10]. The E-D_LSTM model captures long-term dependencies in a temporal series of data, like RUL estimation, by encoding the sequence of wear data to capture its trend for future states and decoding it to foretell future trends. These algorithms, which can learn from sequential inputs with robust performance in predicting time-series outcomes, make them quite effective in applications like tool wear monitoring and RUL prediction, in which accurate prediction of deterioration patterns is critical. This paper also presents the comparative performance analysis of the DeepTool framework through quantitative metrics indicating their ability to predict tool wear and RUL. The paper organisation is as follows: (i) Method details section presents the description of the data collection process, including the setup, data features and operating characteristics (ii) Model Parameters and Performance Metrics section entails the detailed methodology used for the proposed DeepTool framework and its outcomes and lastly (iii) Method validation section presents the performance analysis of the DeepTool framework with similar studies and the related discussion for the same.

## Method details

### Datasets

**Fig. 2:**
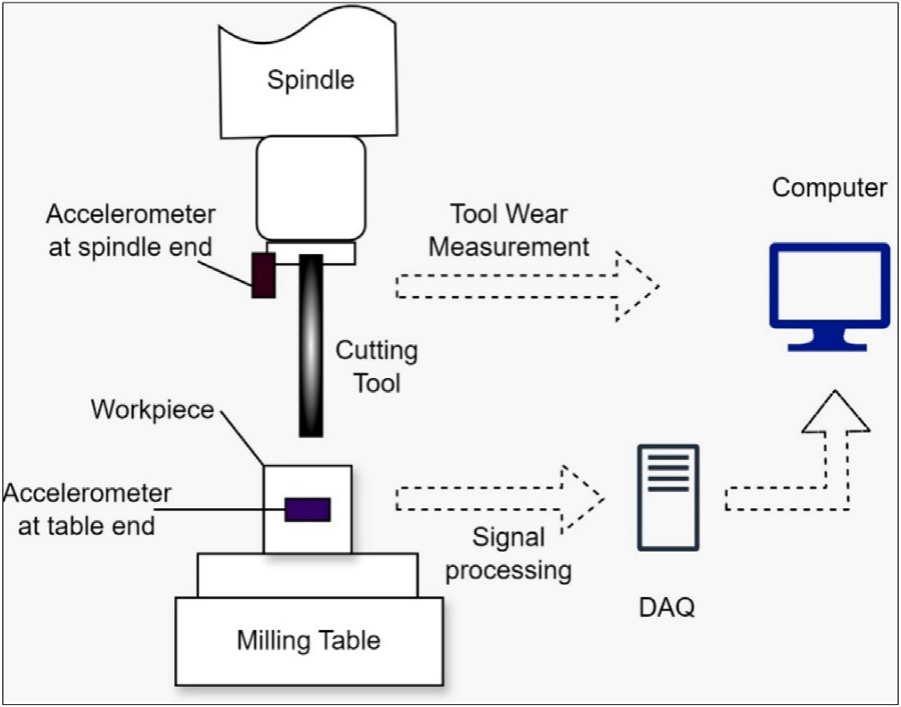
Schematic diagram of the experimental milling setup.

Fig. 2 depicts the schematic diagram of the experimental setup for the data collection process in this study. The experimental setup used to collect data includes a computerised vertical machining control (VMC) system with vertically oriented spindle and table axes to support various cutting tools. The milling tool is fixed in the VMC system, and two high-vibration sensors are used to measure the vibration of the spindle and table. A sample rate of 1000 Hz, corresponding to a motor speed of 1000 rpm, a feed rate of 25 mm/rev, and a depth of cut of 0.25 mm, was maintained throughout the experiment. Tool wear monitoring is recorded for over five minutes (456,000 milliseconds).

An apparatus for data acquisition (DAQ) was used to store the recorded information. The machine's functioning caused signal interference, among other obstacles, but these were overcome by improving the shielding of the sensor wires and positioning the sensors more precisely.

Table 1 presents the operating characteristics for the tool wear prediction experimental setup.

**Table 1:** Tool Wear prediction experimental setup operating characteristics.

Milling cutter:	End-mill Coated carbide inserts
Workpiece:	H13 (Die steel)
Operating Conditions:	Motor Speed: Set at 1000 rpm, balancing tool wear rate and cut quality, enabling accurate wear pattern detection.
	Feed Rate: Maintained at 25 mm/rev to ensure consistent material removal and monitor gradual tool wear.
	Depth of Cut: Set at 0.25 mm to simulate realistic milling with moderate cutting forces for valuable wear data.
	Sampling Rate: Data sampled at 1000 Hz to capture detailed vibration signals, crucial for wear analysis.
	Time Duration: Monitoring lasted 5 min (456,000 ms)

Tool Wear dataset format	Timestamp recorded (in milliseconds)
	Accelerometer sensor signal at table (in Voltage V)
	Accelerometer sensor signal at spindle (in Voltage V)
	Tool Wear value (in mm)

### Model parameters and performance metrics

The original vibration data captured at the spindle end and table end was subjected to the feature engineering procedure to extract indicative time domain and frequency domain characteristics. Feature extraction is crucial to enhance the model's predictive capabilities [11]. The time taken at each instant and the tool's wear are also recorded as a target variable to be estimated. Time-domain and frequency-domain features accurately forecast tool wear progression over a monitored time. Table 2 presents the time-domain and frequency-domain features extracted in this study.

**Table 2:** Time-domain and Frequency-domain features.

Sr No.	Time-domain features	Sr No.	Frequency-domain features
1.	*RMS(Root Mean Square)*	1.	*RMS Frequency*
2.	*Variance, standard deviation, and other statistical parameters*	2.	*Root Variance Frequency*
3.	*Kurtosis and kurtosis coefficient*	3.	*Spectral Skewness*
4.	*Skewness and skewness coefficient*	4.	*Spectral Kurtosis*
5.	*Crest Factor*	5.	*Mean_freq*
6.	*Entropy*	6.	*Magnitude Spectrum*
7.	*Peak value*	7.	*Zero_crossing_rate*
8.	*Shape Factor*	8.	*Power spectral density*
9.	*Clearance Factor*	9.	*Spectral Flatness*
10.	*Impulse Factor*	10.	*Spectral Centroid*

**Fig. 3:**
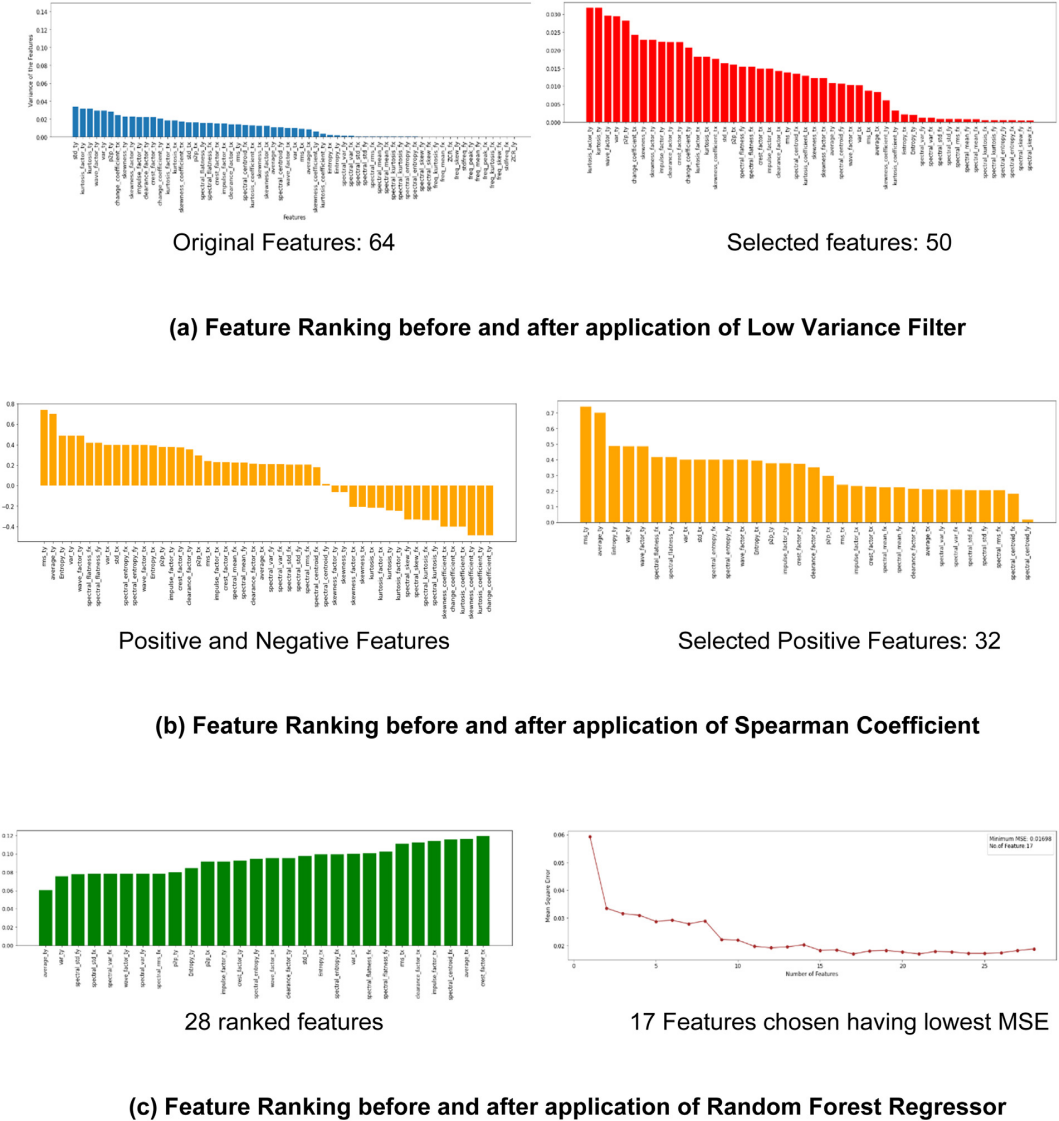
DeepTool feature selection process.

The extracted features are first subjected to an elaborate feature selection step of a low-variance filter [12]. The low variance filter method suggests that features with low variance are unlikely to be informative for predicting the target variable and, hence, are eliminated. Further, the Spearmann Correlation Coefficient is utilised to identify positively correlated feature sets [13]. Finally, the Random Forest Regressor and Recursive Feature Elimination technique (RFE) filter out highly correlated features. RFE focuses on the most important features by removing less important features from the dataset. This improves the interpretation of the model and allows for a more comprehensive understanding of the variables that influence the target variable [14]. Fig. 3 presents the results of the feature selection process of the DeepTool framework.

**Fig. 4:**
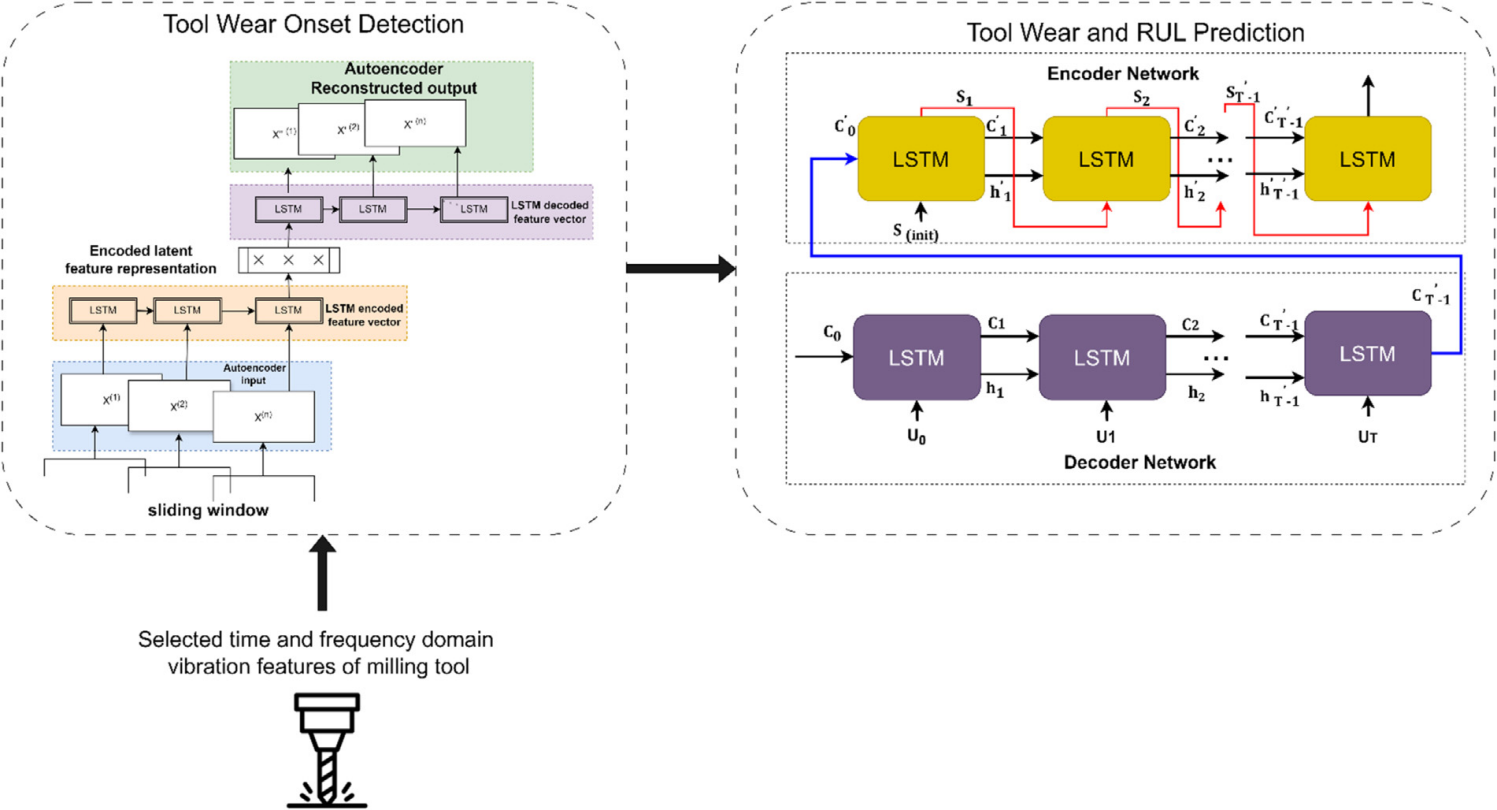
DeepTool framework for tool wear prediction.

The suggested DeepTool framework for tool wear onset detection and prediction is a two-stage process, as shown in Fig. 4. The two stages are as follows:

a. *Tool wear onset identification stage using Autoencoder-LSTM:*

The main emphasis of this study is to identify the onset of tool wear, which acts as a tipping point for further deterioration of the tool. This identification can serve as an input for effective RUL computation of the tool. This stage requires studying inherent patterns in the vibration data with the help of clustering techniques. Clustering helps identify inherent patterns within the tool wear data. Similar wear patterns or behaviours may emerge within clusters, aiding in understanding different tool wear states. Here, k-means clustering with the Silhouette Coefficient approach is used to create normal and abnormal clusters. Clusters produced by K-means clustering may not be equally coherent. The silhouette coefficient aids in the identification of clusters with strong internal coherence and distinct boundaries, making them easier to interpret. This is especially true when interpreting tool wear patterns represented by various clusters. The silhouette coefficient adds credibility to the clustering process by evaluating cluster quality. By ensuring that clusters are formed using precise criteria rather than just visual inspection, this helps to avoid bias in the labeling process.

The Autoencoder-LSTM (AE-LSTM) model is used to detect the initial wear of the tool. While LSTM networks are good at identifying patterns and temporal relationships in data, the autoencoder component of the model is trained to expand and restructure input data by storing important features while minimising reconstruction. The characteristics of sensor data change over time as the device wears out, hence using a reconstruction technique, the Autoencoder model can identify the variance between the inputted data and its reconstructed output.

**Fig. 5:**
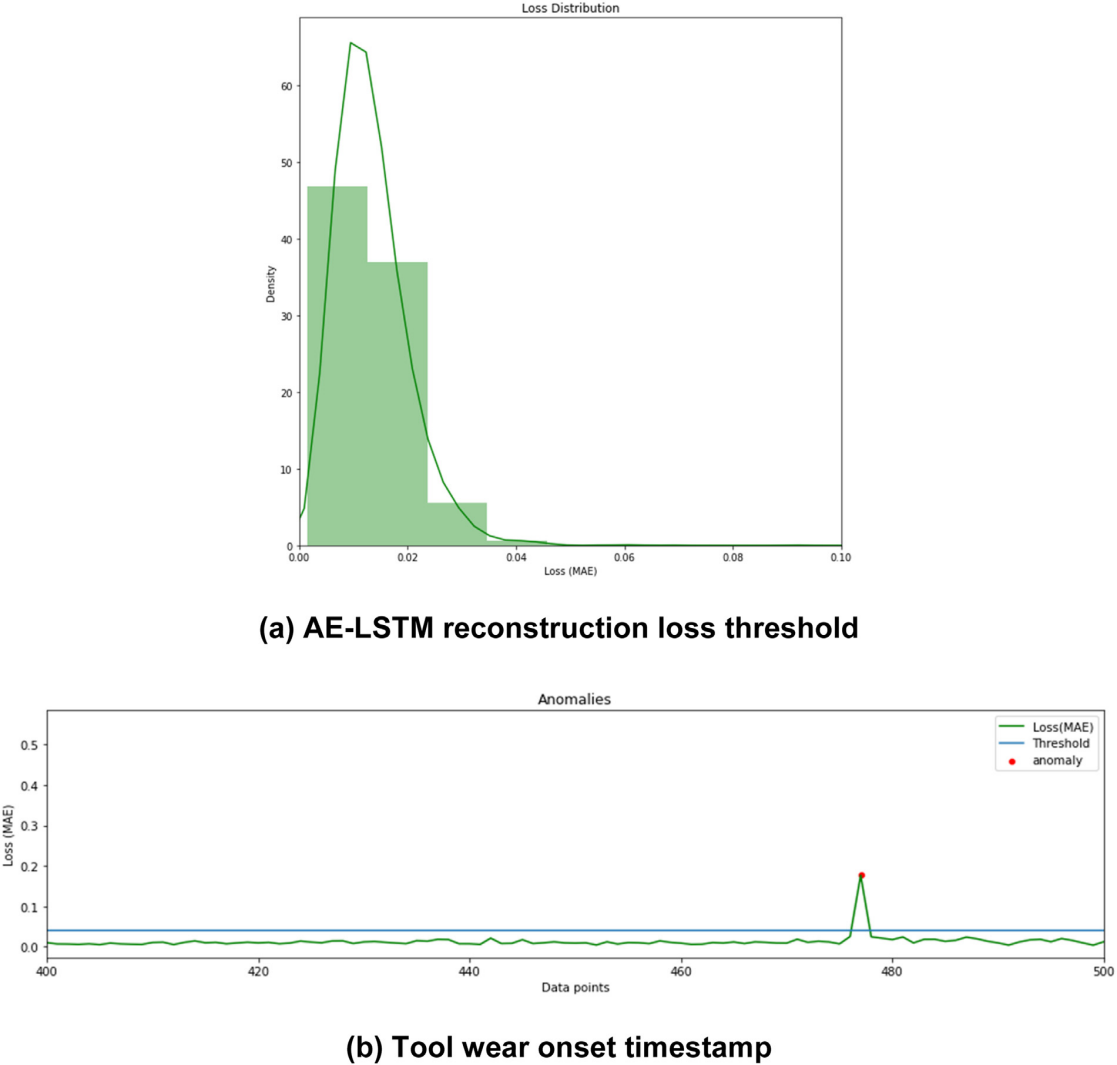
Tool wear onset detection using Autoencoder-LSTM approach.

Fig. 5(a) showcases the Autoencoder-LSTM approach used for identifying reconstruction loss (mae) threshold for anomaly detection. Higher reconstruction loss in this case indicates that the model can't reconstruct abnormal data patterns appropriately, hence indicating the timestamp for the onset of tool wear (Fig. 5(b)).

a. *Tool wear and RUL prediction stage using Encoder-Decoder LSTM:*

To control the sequence-to-sequence tool-wear prediction, an encoder-decoder LSTM model is used, which provides a good estimate of tool wear over time. An encoder processes a sequence of inputs from the sensors and compresses it into a vector representing the state of machinery at that time. Then, the decoder uses the context vector to predict the future sequence of wear-related features or directly estimate RUL. The proposed architecture of the encoder-decoder LSTM enables learning the mapping between past patterns of sensors and the future state, hence being capable of modeling complex temporal dependencies and degradation trends to predict tool wear and remaining useful life in a better way, including situations with non-linearity and multiple factors that affect the wear process [15].

Table 3 presents the model architecture parameters employed in this study. The Encoder-Decoder LSTM architecture processes sequences through an LSTM encoder that captures temporal dependencies into a context vector. This is repeated across time steps with a RepeatVector layer and then decoded with another LSTM layer. The output from there is passed through TimeDistributed Dense layers for final predictions, using ReLU for activation.

**Table 3:** DeepTool framework parameters and model architecture.

Model parameters	Values
activation_function	ReLU
Number_of_epochs	100
optimiser	adam
loss	mse
step_size	10
Number of layers	5 (One LSTM layer, one RepeatVector layer, one LSTM layer, and two TimeDistributed Dense layers
Number of Units	200 units in both LSTM layers, 100 units in the TimeDistributed Dense layer, 1 unit in the last TimeDistributed Dense layer
Model size (MB)	1.942
GigaFLOPS	0.92

**Fig. 6:**
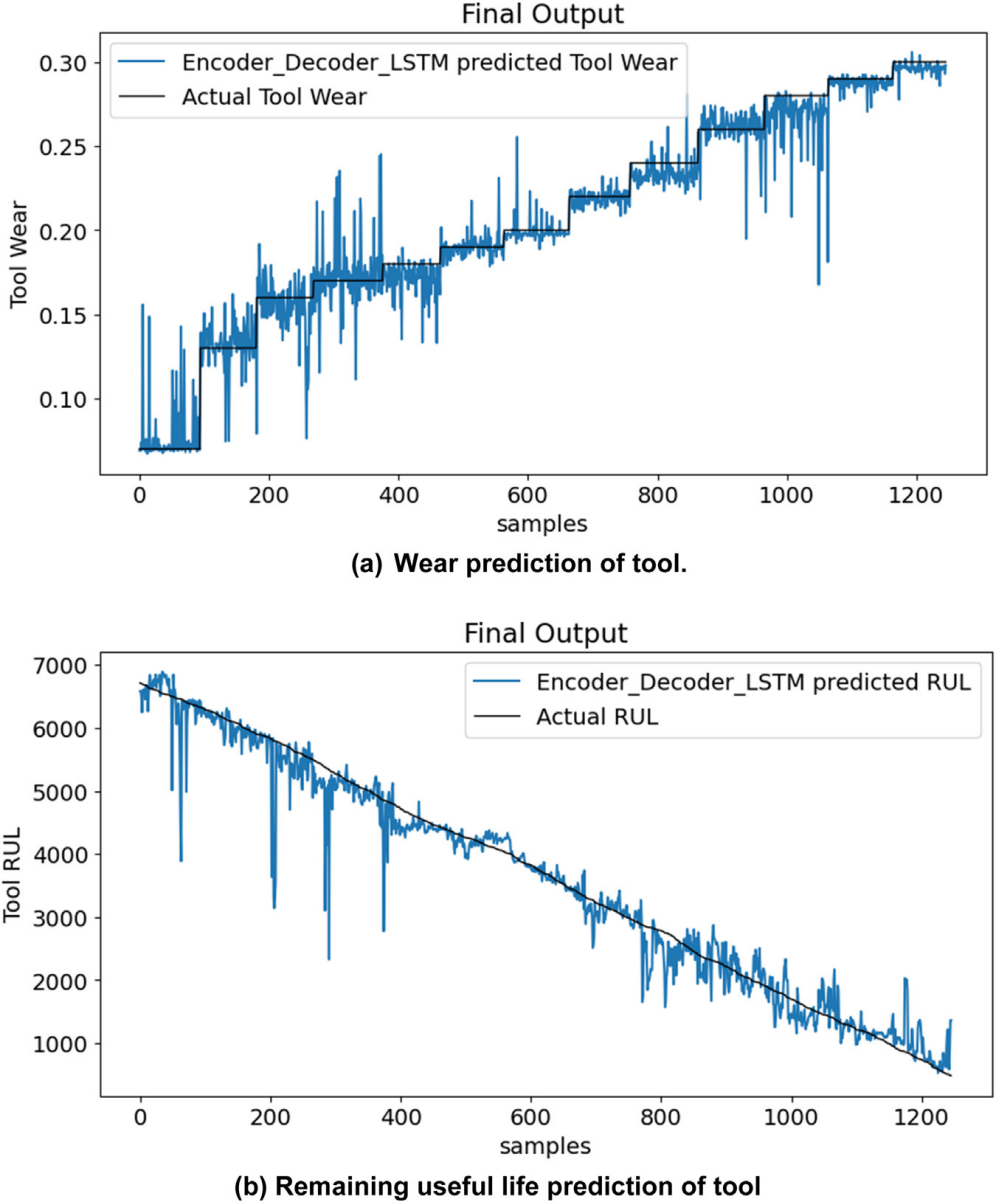
DeepTool framework prediction for milling machinery.

Figs. 6(a) and (b) illustrate the predicted tool wear and remaining usable life of the test set over time. As tool wear progresses toward failure, the tool's remaining usable life reduces progressively. These graphs indicate the progress of tool wear as a gradually increasing curve, thus showing an increase in wear that a tool experiences over time while it continues to run. The RUL curve is trending downward, indicating that less time remains before a tool reaches a failure point.

### Performance metrics

The DeepTool framework performance is analysed w.r.t to regular metrics of R2 score, Root Mean Squared Error (RMSE) and advanced metrics of Mean Absolute Percentage Error (MAPE), Nash-Sutcliffe Efficiency (NSE) and Kling-Gupta Efficiency (KGE) score. MAPE measures the accuracy as a percentage, with lower values indicating better performance; NSE indicates how well the predicted values match the observed values, with a value of 1 indicating a perfect match; and lastly, KGE combines correlation, variability, and bias, providing a comprehensive measure of model performance. Table 4 presents the mathematical formulae for the metrics where

**Table:** 

$y_{i}$	True Value
${\hat{y}}_{i}$	Predicted Value
$\bar{y}$	Mean of true values
n	Number of observations
r	Pearson correlation coefficient between true and predicted values
$\sigma_{\hat{y}}\;and\;\sigma_{y}$	Standard deviations of predicted and true values
$\mu_{\hat{y}}\;and\;\mu_{y}$	Means of predicted and true values

**Table 4:** Performance metrics for model evaluation.

Metric	Mathematical Formulae
MAPE	$\frac{1}{n}\sum_{i=1}^{n}\left|\frac{y_{i}-{\hat{y}}_{i}}{y_{i}}\right|\times \;100$
NSE	$1-\frac{\sum_{i=1}^{n}{\left(y_{i}-{\hat{y}}_{i}\right)}^{2}}{\sum_{i=1}^{n}{\left(y_{i}-\bar{y}\right)}^{2}}$
KGE	$1-\sqrt{{\left(r-1\right)}^{2}+\left(\frac{\sigma_{\hat{y}}}{\sigma_{y}}-1\right)+{\left(\frac{\mu_{\hat{y}}}{\mu_{y}}-1\right)}^{2}}$

### Method validation

Table 5 presents the comparative analysis of recent works in tool wear prediction with the results of the proposed DeepTool framework. Shen Y. et al. [10] created a tool-wear prediction model by combining dynamic smoothing and multi-feature multi-model ensemble with machine learning techniques. SY Lin & CJ Hsieh [11] used ensemble machine learning regression techniques to predict tool wear. In comparison to both works, the proposed DeepTool method has shown superior prediction results in terms of R2 score and RMSE loss values.

**Table 5:** Performance analysis with similar works.

Performance Measure	Shen Y. et al. [16]	SY Lin & CJ Hsieh [17]	DeepTool
R2Score	0.787	0.917	0.951
RMSE	0.00834	0.30053	0.00333
*MAPE*	–	–	17.0973 %
*NSE*	–	–	0.931
*KGE*	–	–	0.90

## Discussions

The DeepTool framework uses time-domain and frequency-domain features for tool-wear onset detection and remaining useful life monitoring using advanced hybrid models of AE-LSTM and Encoder-Decoder LSTM. The following points highlight the main contributions of this study:

i. As the DeepTool framework incorporates time-domain and frequency-domain characteristics during feature engineering, the suggested method outperforms previous studies by getting a higher R² score. Time-domain features record the fluctuations in vibration signal strength as the machine deteriorates, whereas frequency-domain features record short-term information in statistical parameters that represent the machinery's condition over time. These characteristics work together to give a complete picture of the bearing's condition, improving prediction accuracy.
ii. This is further supported by a high value of NSE, indicating that the model effectively represents the variability within the dataset. The KGE goodness of fit value is 0.90, confirming good performance since a well-balanced trade-off between correlation, bias, and variability was reached within the predictions. The close match of NSE and KGE values underlines the model's reliability in the correct dataset prediction.
iii. Also, the study incorporates a sequential feature selection approach using the techniques of Low Variance filter, Spearman Coefficient and Recursive Feature elimination. This approach avoids any bias in feature selection and helps identify the most contributing feature set.
iv. Lastly, the framework utilises the AE-LSTM and Encoder-Decoder LSTM models for tool-wear onset monitoring and prediction. To read the input sequence, the model's encoder is a simple Vanilla LSTM structure, while the decoder might include deep LSTM layers like Stacked_LSTM. The model makes one-step predictions for each layer using the Time-Distributed wrapper function. In comparison to other basic models, this aids the DeepTool framework in achieving a greater level of RUL forecast precision.

## Conclusion

This paper presents an AI-based DeepTool system for milling tool life estimation driven by deep learning that predicts the onset of tool wear and remaining useful life to an accuracy of over 95 % in R2 score. Real-world scenarios are still likely to present huge diversity in the data often that the sensors can produce and further in terms of cutting conditions, thus generally constraining the relative performance that the system may exhibit in practice. In the future, the authors propose to focus on augmenting the dataset with data from a wider range of operating environments to explore advanced models that further increase performance robustness for industrial applications.

## Ethics statements

Not Applicable.

## CRediT authorship contribution statement

**Pooja Kamat:** Conceptualization, Methodology, Formal analysis, Data curation, Writing – original draft. **Satish Kumar:** Conceptualization, Methodology, Writing – review & editing, Resources, Supervision. **Ketan Kotecha:** Writing – review & editing, Resources, Supervision, Funding acquisition.

## Declaration of competing interest

The authors declare that they have no known competing financial interests or personal relationships that could have appeared to influence the work reported in this paper.

## Data Availability

Data will be made available on request.

## References

[1] Zhou Y., Liu C., Yu X., Liu B., Quan Y. (2022). Tool wear mechanism, monitoring and remaining useful life (RUL) technology based on big data: a review. SN Appl Sci.

[2] Cao X., Jing Z., Zhao X., Xu X. (2024). A security-enhanced equipment predictive maintenance solution for the ETO manufacturing. Int. J. Network Manage..

[3] Liao Z. (2024). Review of current best-practices in machinability evaluation and understanding for improving machining performance. CIRP J. Manuf. Sci. Technol..

[4] Bharath H., Venkatesan K. (2023). Study on tool wear mechanism and chip morphology during turning of Inconel 713C by textured inserts. J Manuf Process.

[5] Zhang X., Shi B., Feng B., Liu L., Gao Z. (2023). A hybrid method for cutting tool RUL prediction based on CNN and multistage Wiener process using small sample data. Measurement.

[6] Warke V., Kumar S., Bongale A., Kotecha K. (2024). Robust tool wear prediction using multi-sensor fusion and time-domain features for the milling process using instance-based domain adaptation. Knowl. Based Syst..

[7] L. Zhang et al., “Bioinspired Scene Classification by Deep Active Learning With Remote Sensing Applications”, doi: 10.1109/TCYB.2020.2981480. 10.1109/TCYB.2020.2981480 33635802

[8] Chen Y. (2023). LDANet: automatic lung parenchyma segmentation from CT images. Comput. Biol. Med..

[9] Demir V., Citakoglu H. (2022). Forecasting of solar radiation using different machine learning approaches. Neural. Comput. Appl..

[10] Uncuoglu E. (2022). Comparison of neural network, Gaussian regression, support vector machine, long short-term memory, multi-gene genetic programming, and M5 Trees methods for solving civil engineering problems. Appl. Soft Comput..

[11] Li X., Huang H., Zhao H., Wang Y., Hu M. (2018). Learning a convolutional neural network for propagation-based stereo image segmentation. Vis. Comput..

[12] J. Yu, L. Feng, and J.T. Teši´c, “Data Driven Teacher Attrition Modeling”.

[13] Kamat P., Kumar S., Patil S., Kotecha K. (2024). Anomaly-informed remaining useful life estimation (AIRULE) of bearing machinery using deep learning framework. MethodsX.

[14] Granitto P.M., Furlanello C., Biasioli F., Gasperi F. (2006). Recursive feature elimination with random forest for PTR-MS analysis of agroindustrial products. Chemom. Intell. Lab. Syst..

[15] Coşkun Ö., Citakoglu H. (2023). Prediction of the standardised precipitation index based on the long short-term memory and empirical mode decomposition-extreme learning machine models: the Case of Sakarya, Türkiye. Phys. Chem. Earth, Parts A/B/C.

[16] Shen Y. (2021). Predicting tool wear size across multi-cutting conditions using advanced machine learning techniques. J. Intell. Manuf..

[17] Lin S.Y., Hsieh C.J. (2024). Construction of a cutting-tool wear prediction model through ensemble learning. Appl. Sci..

